# ALK-1-positive inflammatory myofibroblastic tumor of the thyroid complicated by Hashimoto’s thyroiditis: report of a rare case and a literature review

**DOI:** 10.1186/s13000-020-00966-4

**Published:** 2020-05-19

**Authors:** Cheng-fang Li, Xing-long Wu, Jin-jing Wang, Kai Wang, Su-yuan Zhang, Jia-jia Huang, Han-zhong Hu, Hong Zheng

**Affiliations:** 1 Department of Pathology, the Affiliated Hospital of Zunyi Medical University, Zunyi, 563000 Guizhou China; 2 Department of Ultrasonography, the Affiliated Hospital of Zunyi Medical University, Zunyi, 563000 Guizhou China

**Keywords:** Inflammatory myofibroblastic tumor, Plasma cell granuloma, ALK-1-positive, Hashimoto’s thyroiditis, Case report

## Abstract

**Background:**

Inflammatory myofibroblastic tumors (IMTs) of the thyroid are extremely rare soft-tissue tumors. In the literature, IMTs are sometimes called plasma cell granulomas (PCGs) or inflammatory pseudotumors, which often causes ambiguity. To date, 17 cases of PCGs and five cases of thyroid IMTs have been reported. These cases reveal that IMTs of the thyroid are often negative for the anaplastic lymphoma kinase (*ALK*-1) gene. To provide further information on this rare lesion, we present a case of an ALK-1-positive thyroid IMT and a review of IMTs of the thyroid.

**Case presentation:**

A 34-year-old Chinese woman presented with a painless neck mass that had persisted for over a month. Ultrasonography revealed a 4.28 × 2.53 cm^2^ hypoechoic mass, in the left lobe of the thyroid gland. Serum levels of thyroglobulin and anti-thyroglobulin antibodies were high. Subsequently, left lobectomy was performed. Macroscopically, the lesion was a gray-brown nodular mass with a partial envelope. Histologically, two different lesion types were observed. The first lesion showed classic spindle cell proliferation, with spindle cells arranged in fascicles, accompanied by mature inflammatory cells. The other lesion showed a large number of infiltrating lymphocytes, with lymphoid follicles in the remaining thyroid gland, which was atrophic. Immunohistochemical staining showed that the spindle cells were negative for CK19, CyclinD1, Gelectin-3, EMA, CD34, S100, Bcl-2, and STAT-6, but strongly positive for ALK-1, vimentin, and TTF1. CK was focally expressed, and the Ki-67 index was 5%. A diagnosis of IMT was proposed according to immunohistochemical findings and morphology. Hashimoto’s thyroiditis was confirmed according to serum levels of thyroglobulin and anti-thyroglobulin antibodies and morphology. The patient did not receive adjuvant therapy. She remained alive without disease recurrence for 10 months after lobectomy.

**Conclusions:**

IMTs should be considered in the diagnosis when spindle cell proliferation accompanied by mature inflammatory cells is observed, spindle cells are mildly atypical, and myofibroblast differentiation is present in the thyroid. A uniform diagnostic term is crucial to avoid ambiguity. Clinicians and pathologists should be aware of the necessity for long-term follow-up, especially in ALK-positive cases. The therapeutic potential of ALK-1 positivity should be explored further.

## Background

Inflammatory myofibroblastic tumors (IMTs) are potentially malignant or low-grade malignant tumors [1]. IMTs include a heterogeneous group of lesions characterized by the proliferation of myofibroblasts with polyclonal plasma cell, lymphocyte, and eosinophil infiltrates [2]. The tumor exhibits characteristic histological features, with three distinct patterns present in the same lesion: a myxoid, vascular, and inflammatory pattern; a compact spindle cell pattern; and a dense fibrotic pattern [3].

Besides their variable morphologic patterns, IMTs also show a significant molecular heterogeneity, with over ten different anaplastic lymphoma kinase (*ALK*) gene fusion partners identified, all of which contribute to its oncogenic activation [4–8]. ALK fusion partner genes have been identified include NPM, tropomyosin 4 (TPM4), TPM3, CLTC, TFG, ATIC, MSN, RAN binding protein 2 (RANBP2), SE31L1, CARS, and NUMA1. Oscar et al. showed that IMTs harbored kinase fusions involving ALK, ROS1, and NTRK including three novel fusion partners (KLC1, FN1, and RBPMS, respectively) in infantile forms [9]. IMT with rearrangement or translocation of the *ALK* gene and resultant overexpression of the ALK protein, which can be detected by immunohistochemical analysis, reveals a pattern of immunohistochemical reactivity dependent on the fusion partner [9, 10]. Some cases of IMT have a tendency to relapse [11], and IMTs that are ALK-1 positive have been reported to be more aggressive [12, 13].

IMTs are usually found in the lung and upper respiratory tract, and extrapulmonary IMT has also been reported at various anatomic sites, mainly in soft tissues and the viscera [14–18]. In extrapulmonary IMT cases, head and neck lesions account for 14–18% of lesions, whereas location in the thyroid is exceedingly rare [19]. In 2006 the WHO defined IMT as an intermediary lesion with clinical recurrence and malignant potential [1]. IMTs have also sometimes been called plasma cell granulomas (PCGs) or inflammatory pseudotumors in the previous literature. PCG is a pseudotumor-like lesion characterized by polyclonal proliferation of plasma cells and intermingled with lymphocytes and other inflammatory cells in the context of fibrous tissue. This entity has an excellent prognosis with no evidence of recurrence or metastasis [10]. Mostly, the WHO classification of tumors of the lung recommends that PCG should not to be used as a synonym for IMT in the lung [20], and lesions showing a PCG morphology have not been found to show molecular abnormalities.

We found from our search of the English literature using the term IMTs of the thyroid, that there were 22 such reported cases. For all, the diagnosis was PCG and only five cases involved IMTs or inflammatory pseudotumors [13, 21–24]. These reports indicated that IMTs of the thyroid differ from those at other anatomic sites and are often negative for ALK-1. To provide further information on this rare lesion, we report a case of thyroid IMT with ALK-1 positivity and review IMTs of the thyroid. We will discuss the clinical features, diagnosis, differential diagnosis and immunohistochemical characteristics of these rare lesions. Our present report also highlights the diagnostic pitfalls in IMTs of rare anatomical sites.

## Case presentation

**Fig. 1:**
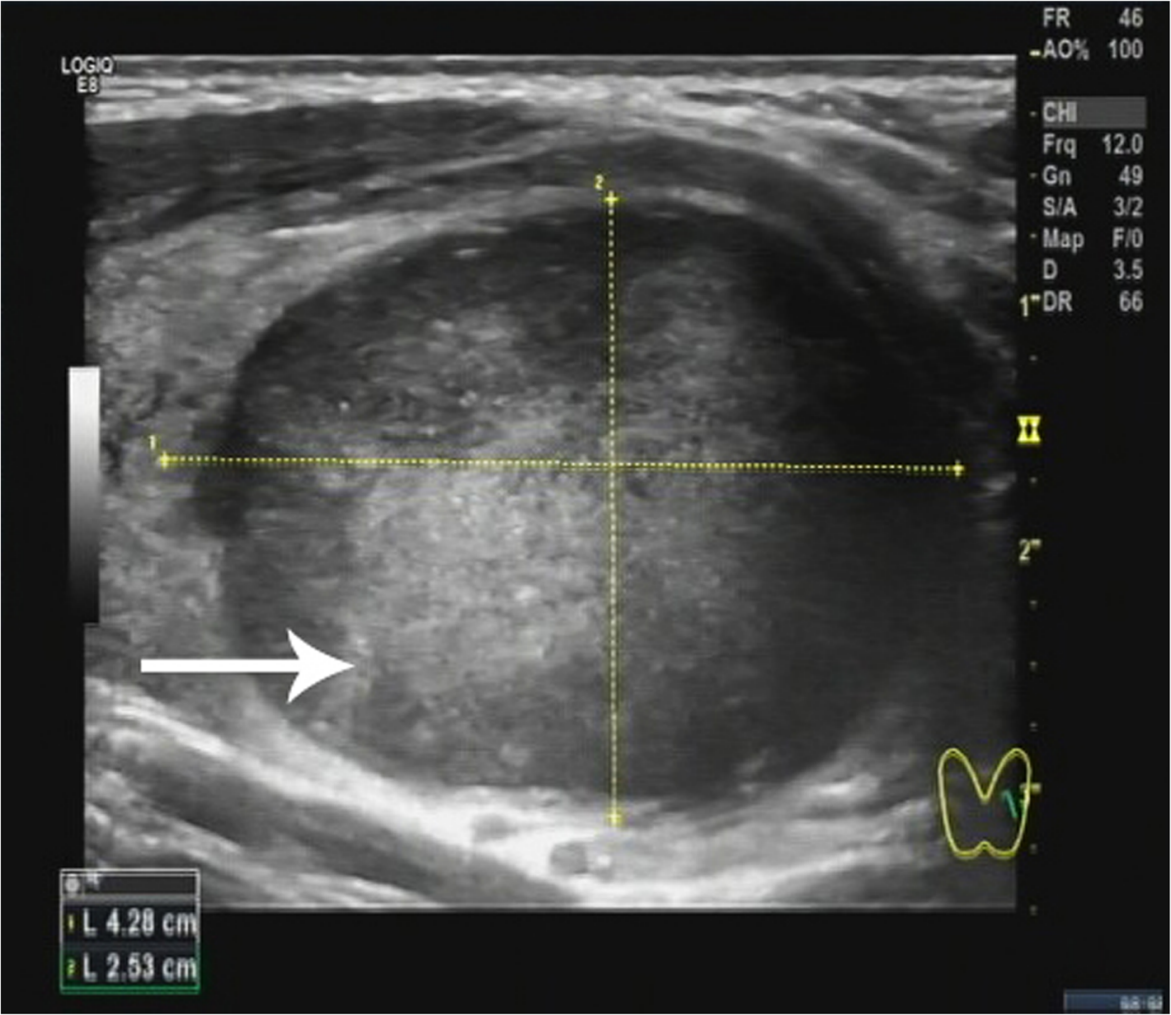
Echographic examination revealed a hypoechoic mass of with clear boundary (The arrow)

A 34-year-old Chinese woman presented with a painless neck mass that had persisted for over a month. Physical examination on admission revealed a solid nodule with a clear boundary, measuring approximately 4 cm, in the left lobe of the thyroid. The serum levels of thyroglobulin were high (180.3 ng/mL, normal reference range: 3.5–77 ng/mL), as were the levels of anti-thyroglobulin antibodies (529.7 IU/mL, normal reference range: 0–115 IU/mL). Other indices of thyroid function were normal. Ultrasonography revealed a hypoechoic mass, 4.28 × 2.53 cm in size, in the left lobe of the thyroid gland. The mass showed clear boundaries and a rich blood flow signal (Fig. 1). Fine-needle aspiration biopsy revealed that it could be an inflammatory hyperplastic lesion, and a left lobectomy was performed subsequently. During surgery, a solid mass, 5 × 3 cm^2^ in size, was observed in the middle of the left lobe of the thyroid. The mass had a smooth surface and clear boundaries, and there was no break in the thyroid capsule.

The greatest diameter of the mass was 4.0 cm. Macroscopically, the lesion was a gray-brown nodular mass with a partial envelope and did not infiltrate the surrounding thyroid parenchyma.

**Fig. 2:**
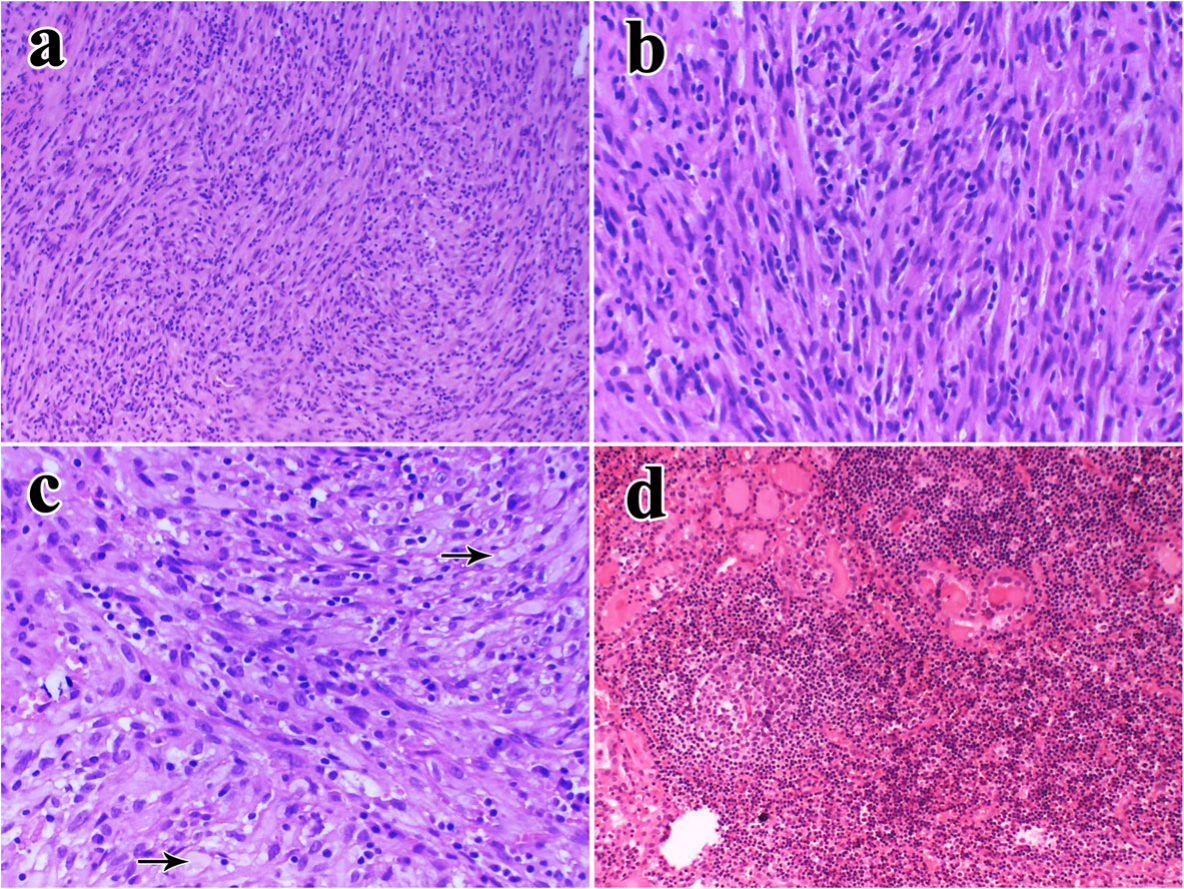
Histology: **a** showed spindle cell proliferation accompanied by uniform distribution of inflammatory cell (100×). **b** These spindle cells were mildly atypia, nuclei was slightly pleomorphic (200×). **c** Some cells were rich in cytoplasm and translucent (The arrow, 100×). **d** In the remaining and atrophic thyroid gland, we found a large number of lymphocytes infiltrate and formed lymphoid of follicles (100×)

Histologically, we found two different lesions. The first lesion showed classic spindle cell proliferation, with spindle cells arranged in fascicles, accompanied by a uniform distribution of mature inflammatory cells such as plasma cells and lymphocytes (Fig. 2a). These spindle cells did not have any mitotic figures, and the nuclei were slightly pleomorphic (Fig. 2b). Focally, the stroma contained abundant hyalinized collagen. Some cells were rich in cytoplasm and were translucent, resembling histocytes (Fig. 2c). The other lesion showed a large number of lymphocyte infiltrates, with lymphoid follicles in the remaining thyroid gland, which was atrophic (Fig. 2d). There was a clear boundary between the two lesions. Careful observation showed that the trapped residual thyroid follicular cells had no characteristic cytological findings of papillary carcinoma.

**Fig. 3:**
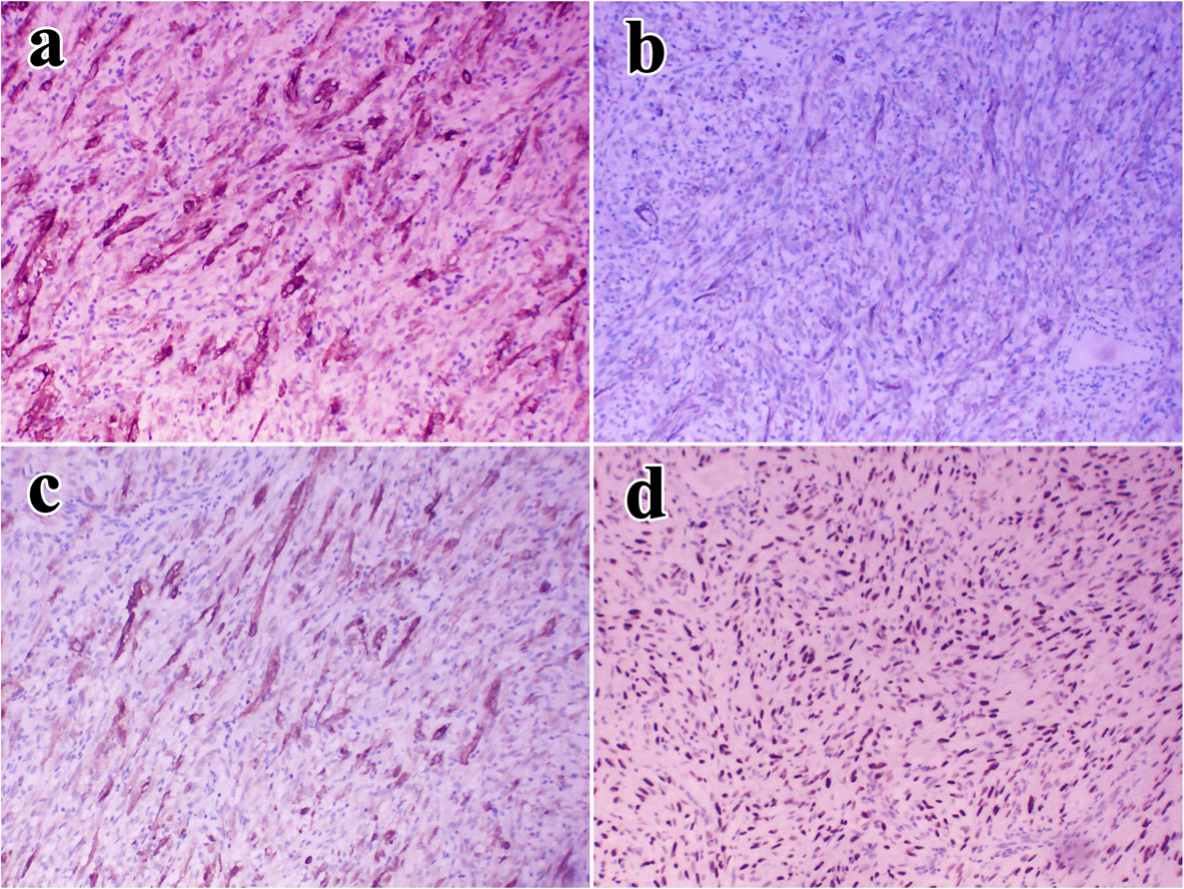
Immunohistochemistry. **a** ALK-1 was diffusely cytoplasmic positive in spindle cells. **b**, **c** The spindle cells were focally reactive to CK (100×) and SMA (100×) respectively. **d** Focal nucleus of spindle cells were reactive to TTF1 (100×)

Immunohistochemical analysis for thyroglobulin (TG), Galectin-3, CK19, CyclinD1, TTF-1, and EMA was performed to rule out the possibility of spindle cell variants of undifferentiated carcinoma of the thyroid. Of these molecules, only TTF-1 was expressed. Furthermore, CD34, S100, Bcl-2, and STAT-6 expression was negative, ruling out the possibility of a solitary fibrous tumor and malignant peripheral nerve sheath tumor (MPNST). Vimentin and ALK-1 were strongly expressed in the spindle cells (Fig. 3a). Cytokeratin (CK) and SMA were focally expressed (Fig. 3b, c), but desmin and EBV expressions were negative. Thorough examination of the thyroid was necessary considering the observed TTF-1 positivity (Fig. 3d), but no characteristics of a well-differentiated thyroid carcinoma were found. Thus, a diagnosis of IMT was proposed on the basis of immunohistochemical findings and morphology, complicated by Hashimoto’s thyroiditis on the basis of the high serum levels of thyroglobulin and anti-thyroglobulin antibodies and morphology.

The patient did not receive adjuvant therapy. Regular follow-ups with physical examinations, blood tests, and ultrasonography were performed. The patient remained well, without IMT relapse, 10 months after lobectomy.

## Discussion

The thyroid gland is an extremely rare site for IMT, and only five cases have been reported in the literature to date (Table 1; to avoid conceptual confusion, PCGs were excluded). Of these five cases, two involved an ALK-1-positive tumor. If we consider the present case, we can state that three of the six reported IMTs (50%) have been ALK-1-positive.

**Table 1:** List of previously reported cases as Inflammatory myofibroblastic tumor

No./ref	Age/Gender	Histology feature	Clinical presentation	HT	IHC	Treatment	Follow up	Echographic examination
1^21^	61/male	fibrohistiocytic	Painless right thyroid mass with swelling	no	Positive: ALK-1 (cytoplasm), SMA, Negative: TTF1, CK, CT, TG, S100, EMA, CD34	Total thyroidectomy	Doing well after 1 year	Hypoechoic with cystic degeneration
2^22^	18/Female	sclerosing	3 cm painless mass of right thyroid	no	Positive: SMA, Vimentin,Desmin, negative: EBV,S100, CD34,ALK-1	Subtotal thyroidectomy	No recurrence 9 months	Hyperechogene nodule with numerous calcification
3^23^	50/female	fibrohistiocytic	0.6 cm right thyroid mass	no	Positive: SMA, CD68 Negative: TTF1, CK 19, CD34, EMA, Bcl2, ALK-1	Total thyroidectomy	Doing well 1 year after surgery	Hypoechoic mass
4^24^	75/male	fibrohistiocytic	1.5 cm painless left thyroid mass for 6 months	no	Positive: Vimentin, MSA, Negative:CK, Desmin, EMA, ALK-1 CycinD1, Bcl-2, CD99	Left lobectomy	Alive without recurrence 10 months	Not provide
5^13^	57/male	fibrohistiocytic	Painless, 4 cm mass in the right and 3 cm in the left with hoarseness	no	Positive: vimentin, SMA, ALK-1((cytoplasm)) Negative: CD34	Subtotal thyroidectomy+ radiation therapy+ steroid therapy	Alive with recurrence and relapse	Hypoechoic mass and heterogeneous echo
6^a^	34/female	fibrohistiocytic	4 cm painless left thyroid mass	Yes	Positive: ALK-1(cytoplasm), Vimentin, CK,SMA, TTF1; Negative: Desmin, CK19, EBV, TG, CD34,Galectin-3, STAT6, Bcl-2, S100	Left lobectomy	Alive without recurrence 10 months	A hypoechoic mass

*HT* Hashimoto’s thyroiditis, *IHC* Immunohistochemistry, ^a^ Present case

An earlier report of thyroid PCG showed a morphological overlap with IMT [25]. However, some studies have suggested that these are distinct lesions and cannot be considered synonyms [21–23, 26].

IMTs are well recognized in the lungs and upper respiratory tract of children and young adults. They show a predilection for the first and second decades of life in other extrapulmonary IMTs [18]. The age of patients with IMT, including that in the present and previously reported cases, ranged widely from 18 to 75 years (average, 49 years), the difference may because of the limited number of identified cases. Our findings are consistent with those of previous reports, whereby both sexes are affected equally [27]. There were no specific symptoms related to thyroid IMT of among the 5 cases that we reviewed, all presented as painless masses, and one patient had a hoarse voice. The present case with IMT exhibited Hashimoto thyroiditis with slightly elevated thyroglobulin and anti-thyroglobulin antibody levels. However, of the five reported cases with IMT, none had no Hashimoto thyroiditis. Four showed hypoechoic nodules on ultrasonography, one showed a hyperechogenic nodule with numerous calcifications, one showed a heterogeneous echo with microcalcifications, which suggested that ultrasound examination could not easily differentiate IMT from thyroid carcinoma and infection thyroiditis.

In terms of morphology, four of the five cases that we reviewed presented a compact spindle cell pattern [24], one case exhibited a sclerosing feature. In the present case, numerous spindle cell proliferations accompanied by a uniform distribution of mature inflammatory cells in the thyroid was observed. A spindle cell neoplasm of the thyroid was considered, and spindle cell variant of papillary carcinoma of the thyroid, anaplastic carcinoma of the thyroid, and solitary fibrous tumor were included in the differential diagnoses. Immunohistochemical study is helpful in diagnosing and distinguishing IMT from other types of tumors. We excluded the possibility of these malignant tumors according to immunohistochemical markers such as thyroglobulin, CK19, MC, and CyclinD1, all of which were found to be negative, ruling out papillary carcinoma of the thyroid. The positive result obtained for TTF-1 may have been because of the residual thyroid epithelium. Moreover, careful examination revealed no trapped residual thyroid follicular cells, which are the hallmark of papillary carcinoma. Although solitary fibrous tumors can have a similar morphology, they are positive for CD34, Bcl-2, and STAT-6 expression, but this was not observed in the present case. Finally, we also excluded the possibility of other mesenchymal tumors, such as MPNST and synovial sarcomas, through negative findings for S100 and EMA expression. It is worth mentioning that such an outcome should not exclude the limitations of our technology. Most IMTs show myofibroblast differentiation with diffusely positive vimentin and focally positive SMA and MSA in spindles, as in our patient. All five of the cases we reviewed showed positive expression for these, indicating the differentiation of myofibroblasts. Besides, expression of WT1 and D240 in the majority of inflammatory myofibroblastic tumors should be considered in the diagnostic work up to minimize misdiagnoses [9, 12].

Genetically, approximately 50% of IMTs harbor *ALK* gene rearrangement with various fusion partner genes [4, 9]. *ALK* immune staining appeared to be determined by its fusion partners [9, 10]. These staining patterns include the following: perinuclear dot-like, diffuse cytoplasmic, granular cytoplasmic, nuclear membranous. ALK-1, which was diffuse and cytoplasmic positive in our case, is expressed in 36–60% of cases, and can be used to differentiate thyroid IMTs from other mesenchymal tumors [25]. Thus, three of the six cases (50%; including the present case; diffuse cytoplasmic in two, granular cytoplasmic in one) involved thyroid tumors positive for ALK, which suggests that IMTs of the thyroid may not be no different from IMTs at other anatomical sites, although there is limited data regarding whether *ALK*-positive tumors differ from *ALK*-negative ones. We did not detect the rearrangement of ALK-1 because of technical limitations. More data need to be collected on these rare cases to clarify their relationship. In addition, abnormal karyotypes of tumor cells, like aneuploidy, were found in 16% of cases [25, 28].

The pathogenesis of IMT remains unknown, but may be related to infection or chronic inflammation [21]. Previous reports have described patients with IMT showing chronic inflammation such as leukocytosis, elevated platelet count, hypergammaglobulinemia, increase of erythrocyte sedimentation rate, and C-reactive protein [29, 30]. In the present case, EBV expression was negative and the blood routine was normal. The patient had Hashimoto’s thyroiditis, and slightly elevated thyroglobulin and anti-thyroglobulin antibodies, suggesting that the immune disorder and infection could have contributed to IMT pathogenesis. These findings were similar to those of PCG of the thyroid in previous reports; however, we did not find any thyroid IMT cases complicated by Hashimoto’s thyroiditis during our review, possibly because of the limited number of cases.

Currently, the mainstream treatment of thyroid inflammatory myofibroblasts is thyroidectomy: total thyroidectomy was performed in three cases, one of which was combined with radiation therapy and oral steroid therapy for the recurrent tumors and the tumor size decreased [13], and subtotal thyroidectomy was performed in remaining three. Recent studies have suggeste that ALK-1 overexpression is associated with recurrence and invasion [31], and extra-pulmonary IMT has been reported to recur [11]. The case we reviewed suffered from local recurrence and metastasis after extensive resection [13]. Recent findings have indicated strong and durable activity of crizotinib in ALK positive IMTs regardless of the tumor location [32, 33]. Thus, we should focus on prognosis and the therapeutic potential of ALK-1 positivity in such tumors. Except for one case with recurrence and metastasis, most patients with IMTs in the thyroid remained alive and well at the follow-up (which ranged from nine to 12 months) without disease recurrence, we recommend continuous follow-up for these patients.

## Conclusions

IMT should be considered as the differential diagnosis in spindle cell lesions of the thyroid, and clinicians and pathologists should be aware of the risk of recurrence and aggressive behavior in rare cases and conduct long-term follow-up. A uniform diagnostic term is crucial to avoid ambiguity. ALK-1 positivity is not uncommon in thyroid IMT, and ALK should be used in the diagnosis and differential diagnosis of such tumors. Further studies should explore the pathogenesis and therapeutic potential of ALK-1 positivity in such tumors.

## Supplementary information

**Additional file 1.** Timeline

## Data Availability

As a case report, all data generated or analyzed are included in this article.

## Abbreviations

IMT: Inflammatory myofibroblastic tumor
PCG: Plasma cell granuloma
ALK-1: Anaplastic lymphoma kinase gene-1
TTF-1: Thyroid transcription factor-1
CK: Cytokeratin
EMA: Epithelial membrane antigen
STAT-6: Signal transduction and transcriptional activator 6
BCl-2: B-cell leukemia/lymphoma 2 gene;
TG: Thyroglobulin
MPNST: Malignant peripheral nerve sheath tumor
