# Supplementary material for: ALK-1-positive inflammatory myofibroblastic tumor of the thyroid complicated by Hashimoto’s thyroiditis: report of a rare case and a literature review
Source: Diagn Pathol. 2020 May 19;15:58. doi: 10.1186/s13000-020-00966-4 (PMC7236920; doi:10.1186/s13000-020-00966-4)
Supplement: Supplementary file 1 — Additional file 1. Timeline [file 13000_2020_966_MOESM1_ESM.docx]

Timeline

| Timeline | Relevant data |
| --- | --- |
| Day 1 | Admission and physical examination: a solid nodule with a clear boundary measuring approximately 4cm in left lobe of thyroid |
| Day 2 | The serum levels of thyroglobulin were high (180.3 ng/ml, normal range: 3.5-77 ng/ml), the levels of thyroglobulin antibody were 529.7 IU/ml (normal range: 0-115 IU/ml). Other indexes of thyroid function were normal.  The ultrasound image revealed a hypoechoic mass of 4.28cm×2.53cm in the left lobe thyroid gland.  Fine-needle aspiration biopsy revealed a possible inflammatory hyperplastic lesion. |
| Day 7 | Left lobectomy without any adjunctive treatment |
| Day 13 | Pathological diagnosis：Inflammatory myofibroblastic tumor complicated with Hashimoto’s thyroiditis. Immunohistochemistry: CK, SMA, Vimentin, TTF-1 and ALK-1 were positive, and TG, CK19, Galecitn-3, EMA, S100, Bcl-2, STAT6, CD34, EBV and Desmin were negative. |
| Day 15 | No any discomfort after surgery, discharged from hospital. |
| Month 6,10 | Return to hospital for reexamination by the neck ultrasound, the patient was alive without disease recurrence |
